# Surgery Training and Simulation Using Virtual and Augmented Reality for Knee Arthroplasty

**DOI:** 10.7759/cureus.28823

**Published:** 2022-09-06

**Authors:** Pooja Mandal, Ratnakar Ambade

**Affiliations:** 1 Medicine, Jawaharlal Nehru Medical College, Datta Meghe Institute of Medical Sciences, Wardha, IND; 2 Orthopaedics, Jawaharlal Nehru Medical College, Datta Meghe Institute of Medical Sciences, Wardha, IND

**Keywords:** surgical training, simulation, knee arthroplasty, rehabilitation, augmented reality

## Abstract

A range of extended reality technology integration, including immersive virtual reality (IVR), augmented reality (AR), as well as mixed reality, has lately acquired favour in orthopaedics. The utilization of extended reality machinery in knee arthroplasty is examined in this review study. Virtual reality (VR) and AR are usually exercised together in orthopaedic surgical training as alluring training outside of the operation theatre is acknowledged as a good surgical training tool. The use of this technology, its consequences for orthopaedic surgeons and their patients, and its moral and practical issues are also covered. Head-mounted displays (HMDs) are a potential addition directed toward improving surgical precision along with instruction. Although the hardware is cutting-edge, substantial effort needs to be done to develop software that enables seamless, trustworthy integration into clinical practice and training. Remote virtual rehabilitation has drawn increasing attention in recent years, and its significance has increased in light of the recent outbreak of the COVID-19 epidemic. Numerous medical sectors have shown the benefits of telerehabilitation, gamification, VR, and AR. Given the rising demand for orthopaedic therapy and its rising costs, this is a requirement. A remote surgeon can impart knowledge without being present, by virtually placing his or her hands in the visual field of a local surgeon using AR technology. With the use of this innovation, orthopaedic surgery seems to have been able to participate in the telemedicine revolution. This technology may also have an impact on how surgeons collaborate and train for orthopaedic residencies in the future. Volatility in the HMD market will probably stall improvements in surgical education.

## Introduction and background

One of the four most common chronic musculoskeletal disorders, according to the World Health Organization, is osteoarthritis [1]. Patients with advanced osteoarthritis of the knee may benefit from total knee replacement (TKR), a standard surgical procedure [2]. Physical therapy is a crucial component of the healing process, with routine and specialised regimens meant to restore function and ease the pain. Researchers and doctors are becoming more interested in the possibilities provided by virtual reality tools (VRT) in this area. The application of surgery, neuroscience, psychology, physical therapy, and rehabilitation in the field of healthcare has increased [3]. With ongoing technological advancements, there has been substantial interest in utilising augmented reality (AR) as well as heads-up displays (HUD) in the field of surgery. These advancements in orthopaedic surgery have aimed to improve patient care while also providing advantages to the surgical team [4].

In orthopaedic surgery, the usage of AR has been described as early as 2007. Ortega et al. evaluated the effects and possible advantages of utilising a HUD during spine surgery. According to further research, advantages may include an increase in the surgeon's focus on the surgical field, a decrease in potentially dangerous radiation exposure, and a reduction in surgical field barriers, additionally through services such as rehabilitation that are provided outside of hospitals [5,6]. There is still disagreement over the best surgical course of action for isolated medial compartment osteoarthritis of the knee. Despite the possibility of a high tibial osteotomy, the majority of patients receive care through either a unicompartmental knee arthroplasty (UKA) or a total knee arthroplasty (TKA) [7]. Both supervised and unsupervised activities are currently used as rehabilitation treatments, but technological advancements are bringing up new possibilities in this area. For the rehabilitation of orthopaedic patients, virtual reality (VR), AR, gamification, and telerehabilitation are interesting. By tricking the patients' brains into thinking they are somewhere other than their actual location, VR and AR hope to improve patient outcomes. In VR, the patient engages with a virtual setting to mimic real-world action. The downside of this technology is that it makes it impossible to identify actual threats that can harm people. In AR, virtual and physical worlds collide, and the patient is aware of any potential risks [8]. Patient-specific instruments have been brought into clinical practice to save operating time, recreate the mechanical axis determined prior to surgery using MRI imaging, and adapt the implant to the patient's deformity. In more recent times, MR and AR have expanded these technologies' potential uses to include operating rooms.

The precision of component location in hip and knee arthroplasty has been proven to be improved using AR-based navigation technology [9]. The goal of this narrative review is to explore how immersive virtual reality (IVR), AR, along with MR machinery, are used during knee replacement. We start by briefly describing its applications in pre-surgical and intraoperative localization, and then we go into great depth on how it might revolutionise current surgical education and training. To show how useful a cutting-edge IVR simulator is for instructing trainees in knee arthroplasty techniques, we also present a sample workflow.

## Review

Knee arthroplasty

Arthroplasties are surgical procedures where a joint is replaced with unnaturally made materials. A total arthroplasty entails replacing all the affected joint surfaces, whereas a partial replacement merely replaces some or all the affected surfaces. The most often replaced joints are knee joints. Symptomatic osteoarthritis and fracture of the neck part of the femur are the two most representative reasons for knee replacement surgery. Patients who need their first hip or knee replacement owing to osteoarthritis are typically between 60 and 70 years old. The most common reasons for joint replacements, which cause joint surface degradation via the wearing of the cartilage coating, include degenerative diseases such as osteoarthritis, traumas, and other modifications in the osteoblasts and connective tissue components. In some circumstances, they can cause a decline in quality of life with a lifelong impairment, persistent pain, and limited mobility of the afflicted joint. For the patient to continue their full range of daily routines and prevent subsequent problems, restoration with an artificial material becomes crucial if these symptoms do not go away.

Osteoarthritis

Knee arthroplasty is often performed in the case of osteoarthritis. Risk factors include age and sex, hereditary, biomechanics, and proinflammatory components. Body weight, osteoporosis, cardiovascular disease, and metabolic disorders are some variables that might negatively affect cartilage metabolism. Local consequences may result from trauma, circulatory problems, inherited abnormalities, and excessive stress on just one side of the joint [10]. An imbalance in cartilage metabolism, where catabolic activities predominate, is a hallmark of osteoarthritis. New, less durable cartilage tissue first develops due to cartilage degradation. As a result, joint functionality is reinstated, but the joint has decreased strain resistance. The cartilage tissue may entirely degrade over time, exposing the underlying bone that thickens the joint.

Indications of knee arthroplasty

Modern research confirming favorable outcomes with UKA in younger patients, obese people, patients with patellofemoral dysfunction, and those who are very active has substantially enlarged the original stringent inclusion criteria of Kozinn and Scott [11].In all patients with anteromedial OA who have a correctable deformity, intact knee ligaments, and preserved range of motion in the knee but less than 15 degrees of contracture, medial UKA may be an option (Table 1). UKA should be avoided in patients with inflammatory arthropathies, and those who have had a high tibial osteotomy in the past should utilize it with caution. Because partial thickness loss causes inconsistent pain alleviation and has a six times greater likelihood of revision, patients should have total thickness cartilage loss and avascularity. Although peripheral T-cell lymphoma (PTCL) has comparable clinical results, it was associated with necrosis [12].

**Table 1:** Patient's characteristics HKA: hip knee ankle; MPTA: medial proximal tibial angle; mLDFA: mechanical lateral distal femoral articular angle; AMA: American Medical Association; JLCA: joint line conversion angle.

Patients	Age (y)	Gender	BMI (kg/m^2)^	HKA (°)	MPTA (°)	mLDFA (°)	AMA (°)	JLCA (°)
Patient 1	70	M	35.13	176	95	90	4	4
Patient 2	78	F	34.94	174	82	91	6	5
Patient 3	77	F	27.27	183	88	87	5	6
Patient 4	79	F	30.42	172	91	94	3	5
Patient 5	75	F	34	177	80	92	6	2

AR, VR, and simulation in orthopaedic education: a historical overview

Surgical Education and Practice in Knee Replacement Surgery

The degree to which a simulation qualitatively matches the model or fidelity can vary widely. Despite the likelihood, as relatively high simulations may benefit students more, studies have demonstrated the benefits of utilizing minimal simulators in surgical training. For instance, it has been discovered that Fundamentals of Laparoscopic Surgery (FLS) (Society of Endoscopic and Gastrointestinal Surgeons, Los Angeles, CA) transfers skills to the operating room more effectively than a comparable high-fidelity general surgery simulator [13]. In orthopedic surgery, various VR simulations can be employed with usually positive outcomes. The first of these was a simulator for knee arthroscopy with haptic feedback, developed in the late 1990s with support from the American Board of Orthopaedic Surgery. Since then, numerous arthroscopy simulation systems for the shoulder and knee have been created [14]. In addition to the actual interventions, AR in surgical training has many potential applications, including animal, carcass, high-fidelity, and low-fidelity simulations. AR has frequently been utilized in endoscopy, laparoscopy, and arthroscopy to teach surgeons complex procedures in small surgical spaces. AR training in FLS trainers accelerated the learning curve for beginners learning intracorporeal suturing methods [15]. Using AR technology, complicated surgical procedures on cadavers can now be remotely instructed [16]. The use of AR in orthopedic training is yet largely unexplored. The attending orthopedic surgeon might guide a resident from a distant station in a viewing room till the surgeon's involvement in the operating theatre is required. The remote AR workstation included a computer that digitally superimposed the surgeon's hand onto the surgical area display the resident was viewing, a camera, a phone for a two-way teleconference, and a display showing the arthroscopy image. Additional research is required to determine whether AR can help residents learn more quickly, particularly as competency-based milestones are incorporated into resident training [17].

Preoperative Strategy

Proper component placement is crucial for maximizing implant life in knee arthroplasty. In the past, surgeons would often strive for a mechanical center that would be less than 3° from neutral to eliminate premature failure, notably on the tibial side [18]. While performing complete knee arthroplasty, coronal alignment has to be taken into account in addition to the femoral element's range of motion, femoral flexion, and tibial inclination in the sagittal plane polyethylene size, as well as soft tissue balance. CT imaging may be the best method for identifying these anomalies before surgery because the parameters as mentioned earlier, are multidirectional [19]. The most reliable metrics for evaluating the usefulness and advantages of surgical simulators are face, content, construct, and concurrent validity. The appropriateness of the simulator's psychomotor fidelity and the variables tested, respectively, are judged according to face and content validity. The simulator's ability to differentiate between beginners and specialists is objectively measured by construct validity. The term "concurrent validity" refers to the relationship between the variables measured and performance in the real world [20].

AR-Based Intraoperative Navigation

In TKA, computer navigation has increased alignment precision, and some authors have hypothesized that this may eventually increase implant survival rates [21,22]. The rate of revision for contemporary knee prostheses is minimal. Therefore, if there is a benefit, it is likely that finding it will require extensive follow-up and a large sample size. Age substantially impacts the revision rates after TKR, with younger age groups experiencing more excellent revision rates [23]. The innovative AR surgical platform eliminates the need for heavy external detection equipment or reflecting markers by using small detectors with an inherent detecting mechanism attached to the femur and tibia by pins implanted inside the surgical site. Using patient-specific CT imaging, a personalized preliminary plan is created. A unique methodology allows for identifying ligament insertion and origin to track ligament balance during intraoperative navigation.

There are many benefits of AR-based navigation over existing navigation systems, despite the requirement for preoperative CT imaging, which adds to the patient's radiation exposure and demands time for scan acquisition. Because AR navigation tools are more compact and more straightforward than traditional computer-assisted systems, they might not require the significant capital expenditures and ongoing maintenance costs that come with them. The small and portable type of enrollment and monitoring equipment will also significantly reduce the footprint in the operating room as ambulatory TKA becomes more popular as a way to lower facility costs, making this innovation quite logistically feasible for ambulatory surgery centers (ASC) [24].

IVR for training in surgery

Many orthopedic simulators and task instructors have been developed and studied since the first VR knee arthroscopy simulators were released in the 1990s [14]. With the help of contemporary simulators, students can practice techniques including arthroscopy, fracture reduction, sawing, and drilling. There is a need for clearly intended programmes to impart technical information even outside the operating room environment because of rising resident training costs, decreasing work hours, and moral issues about patient care [25]. Dissection models have long been considered to be the gold in surgical teaching due to the apparent advantage of improved accuracy with regard to tissue feel. However, there isn't much, high-level evidence that they can transition from acquiring an ability to having a technical aptitude in actual operating contexts. Students need to practice their surgical, diagnostic, and planning before operating, as well as use their judgment skills, outside of the operation theatre with the aid of VR-based simulators. One of the main advantages of VR simulation over real-world modeling is the capability for beginners to obtain instant, critical praise, recognition, and support without the requirement for in-person expert coaching [26]. VR in orthopedics demonstrates the reliability and dependability of simulations for hip and intraarticular fracture repair, as well as knee, hip, and shoulder arthroscopy. The applicability of these methods in actual assessment and training for more experienced surgeons may be constrained by these findings [20].

Surgical Simulation in IVR for a TKR

Throughout the knee replacement procedure, a complex combination of technical (saw control and soft tissue managing), integrated (retractor configuration, patellar resurfacing), intellectual (tibial and femoral alignment and rotation, soft tissue balance, and implant size), and technical (integrated) skills are needed. The accuracy of implants and surgical problems have been linked to reduced volume and incompetent doctors [27,28]. Orthopedic residency training frequently involves original knee arthroplasty, while revision surgeries are somewhat less prevalent. Failure rates after TKA are predicted to be 1% annually, with sterilized loosening being responsible for 22.8%-31.2% of failed cases [29]. Despite this increased demand, specialized fellowships or individualized practice are frequently used to acquire the technical and intraoperative decision-making abilities necessary in revision arthroplasty. IVR, on the other hand, offers a way for medical professionals to enhance their skills in revising TKA by simulating a variety of distinct clinical settings, which shifts the learning curve [30].

Drawbacks of IVR

The visual quality, level of presence, cyber-sickness, haptic realism, device-related concerns (including battery life and wireless technologies), and cost considerations are only a few of the drawbacks of immersive visual reality systems. Computing power and software optimization are required to produce complex audio-visuals as well as second-by-second haptic output of IVR systems. As a result, there are differences in the IVR systems that are currently accessible with regard to experience quality, immersion and presence, and learning potential [31,32]. The constellation of signs and symptoms brought on by IVR exposure has been labeled as cybersickness. Although there isn't a direct link between cybersickness and presence or immersion, self-motion in reality while stationary in the virtual world, visual display factors (refresh rate: 20 fps or visual field: 40 degrees), and turbulent interactions in a virtual environment are risk factors for cybersickness [33]. The use of IVR as a practical and cheap alternative to training programs in surgical practice has been proposed. Future generations of hardware are anticipated to be significantly less expensive than earlier incarnations as technology advances.

Future of AR and VR

The intraoperative usage of HMDs and their function in clinical treatment have not yet been thoroughly proven, despite the popularity of AR for surgical navigation. If larger clinical studies show benefits over present practice, HMDs may shortly be used to exhibit critical data, photos, and even fluoroscopy imaging in the surgeon's visual field. More sophisticated tracking software needs to be developed to maximise the usefulness of these devices within the operating theatre. The solitary solution is electromagnetic surveillance, which does not require a line of vision, although the techniques are frequently limited by interference from metallic instruments [34,35].

## Conclusions

Both surgical care and surgical education are increasingly relying on VR and AR. Although several studies have exemplified that VR enhances skills even outside the surgery room, it is difficult to determine how effectively these abilities transition to the clinical context. AR techniques for orthopaedic instruction and skills have shown significant promise in terms of enhancing planning before operating, offering intraoperative assistance, along with increasing the precision of surgical procedures. Moreover, these instruments are projected to become more prevalent both in training and clinical services as the equipment's ability to replicate the operative setting improves. Currently, AR is mostly employed for simulated and intraoperative navigation. Most crucially, IVR technology has shown that it has the power to completely transform contemporary surgical education and cost-effectively increase surgical performance, eventually improving patient outcomes. Future uses of IVR may also involve the potential for remote mentorship to address the global access gap to high-quality surgical training. As vital as it is for knee surgeons to deliver affordable treatment, it is also important to continue defining how new technology improves patient outcomes and determine whether or not all patients may benefit from it. As a result, as a profession, we must continue to do and publish high-quality research that identifies which innovations are real advancements and which are simply new ways of performing an operation.
